# Acute Effects of Self-Selected Music Intervention on Golf Performance and Anxiety Level in Collegiate Golfers: A Crossover Study

**DOI:** 10.3390/ijerph17207478

**Published:** 2020-10-14

**Authors:** Hung-Tsung Wang, Hsia-Ling Tai, Chia-Chen Yang, Yung-Sheng Chen

**Affiliations:** 1 Graduate Institute of Sports Training, University of Taipei, Taipei 11153, Taiwan; justin580817@gmail.com (H.-T.W.); jazellayang@hotmail.com (C.-C.Y.); 2 Department of Physical Education, University of Taipei, Taipei 10048, Taiwan; 3 Department of Exercise and Health Sciences, University of Taipei, Taipei 11153, Taiwan; yschen@utaipei.edu.tw

**Keywords:** golf swing, golf putting, pre-exercise music, simultaneous music, psychology, autonomic nervous system

## Abstract

Music has been reported as a positive intervention for improving psychophysiological conditions and exercise performance. However, the effects of music intervention on golf performance in association with psychophysiological responses have not been well examined in the literature. The purpose of the study was to investigate the acute effects of self-selected music intervention on golf swing and putting performance, heart rate (HR), HR variability (HRV), and anxiety. Twenty collegiate golfers voluntarily participated in this study (age = 20.2 ± 1.4 years, height = 171.7 ± 8.0 cm, body weight = 69.5 ± 14.6 kg, golf experience = 7.5 ± 2.1 years). A cross-over and within-subject design was used in this study. Participants performed a non-music trial (T1), pre-exercise music trial (T2), and simultaneous music trial (T3) in a randomized order with 48–72 h apart. The participants were attached to a HR monitor to record the HR and HRV during the measurement. The golf swing and putting performance was assessed by using the Golfzon golf simulator system. The state-trait anxiety inventory-state questionnaire (STAI-S) was used to evaluate anxiety state. All measurements were taken during baseline (phase one) and after resting or music intervention (phase two). Repeated measurement of analysis of variance (ANOVA) and Cohen’s effect size (ES) were used for statistical analyses. The results show no significant differences in golf swing and putting performance (*p* > 0.05). However, significant decrease in STAI-S score was found in T2 (*p* = 0.047, ES = 0.32). A significant increase in the standard deviation of normal R-R interval (SDNN), low-frequency power spectrum (LF), standard deviation of along the line-of-identity (SD2) in T2 and T3 were observed (*p* < 0.05). In conclusion, a single pre-exercise or simultaneous self-selected music intervention contributes minor effects to golf performance in collegiate golfers. The positive benefits of self-selected music intervention on the psychological condition and cardia-related modulation while practicing golf is warranted.

## 1. Introduction

Golf is recognized not only as a leisure activity for the general population but also as a competitive sport for elite golfers. Golf skills include swing and putting performance, which is categorized as closed-chain kinetic exercise. To complete a shot, golfers rely on the coordination of the head, upper limbs, trunk, and lower limbs to control the sophisticated movements and coordinate the body segments in order to hit the ball to approach the target. The full swing is considered the most complicated movement, requiring excellent dynamics of the entire body and control of the velocity of swing performance during the golf swing cycle [1,2]. In contrast, the putting stroke is a sophisticated action that requires stability of motor performance and mental status for accuracy of shots [3]. Despite the skills acquisition and kinetic movements of the body segments being different for controlling a full swing and putting, it all relates to the effectiveness of neuromuscular controls and coordination in psychophysiological mechanisms for rotational biomechanics of the golf shots.

Music intervention might provide physiological and psychological recovery via respiratory rhythms with music tempo. Music has been documented as an optimal modality to enhance strength performance [4,5,6], swimming [7,8], cycling [9,10], running [11,12,13], circuit exercise [14,15], Wingate anaerobic test [16,17,18,19], and skills acquisitions [20,21]. This evidence demonstrates that music intervention is a useful tool to alter psychological responses and fatigue-related symptoms in association enhancing exercise performance and recovery status. Music intervention might have benefits on physiological and psychological recovery via respiratory rhythms with music tempo [22]. The effectiveness of music on brain activities and its relations to perceptual sensation during various exercise protocols has been demonstrated in a series of studies conducted by Bigliassi et al.’s research group [6,10,12,23]. The cerebrum plays a major role in regulating the neurophysiological mechanisms in response to music stimuli. It has been demonstrated that increases in cerebral activities in the temporal lobe, frontal lobe, insular cortex, and limbic system were found in response to music stimuli [24].

Elite athletes frequently experience physiological constraints and mental stress in regular training and competitions. Intense sports training and competitions might contribute to fatigue-related syndromes and the heavy burden of psychophysiological response for the athletes, consequently impairing exercise performance. From a practical point of view, athletes prefer to listen to music before training sessions due to mental and perceptual issues (i.e., relief of stressors and competition-related anxiety) [25]. This pre-event process can help athletes to prepare for the game through rehearsing association imaging and relaxation skills [26]. It has been demonstrated that pre-task music intervention could improve power and strength performance [27]. For example, Biagini et al., [5] reported that university students improved deep squat jump performance (i.e., velocity of push off, velocity, rate of force development) and reduced psychological fatigue score after listening to preferable music. In contrast, simultaneous music could contribute to a complex of cognitive function during motor performance. For example, Stork, Kwan, Gibala and Martin [19] demonstrated that simultaneous music intervention could enhance peak and mean power of the Wingate anaerobic test and perceived enjoyment during repeated bouts of 30 s all-out anaerobic performance. Another study to address the notion of simultaneous music intervention to improve 400 m sprint performance has been reported by Simpson and Karageorghis [28]. Thus, implementation of music intervention before and during exercise contributes to improvement in short-term exercise performance.

To our knowledge, only one quantitative study [29] and one qualitative study [30] have examined the effect of music intervention on golf performance. However, information is still limited to us regarding the optimal strategy for music use in golf training or competition. For example, comparison of music intervention before and during the golf performance is not available in the literature. Since the psychological status [31] and cardiovascular responses [32] are associated with motor behaviors and music, it is essential to investigate the contribution of music intervention among these factors. In light of the above-mentioned components, the primary purpose of this study was to investigate the acute effects of pre-exercise and simultaneous self-selected music interventions on golf swing and putting in collegiate golfers. The secondary purpose was to determine anxiety, the heart rate (HR), and HR variability (HRV) during pre-exercise and simultaneous music tasks. It was hypothesized that golf swing and putting performance would significantly improve during pre-exercise and simultaneous music trials. It was also hypothesized that anxiety level would be significantly lower and the HR and HRV would recover faster in pre-exercise and simultaneous self-selected music trials than that of non-music trials.

## 2. Materials and Methods

### 2.1. Experimental Approach to the Problem

This study investigated the acute effects of self-selected music intervention on closed-chain motor skills of golf performance in addition to anxiety and autonomic nervous functions. A cross-over and within-subject design was used to investigate the acute effects of self-selected music intervention on golf swinging and putting performance, anxiety level, HR, and HRV indices. The baseline measurement (phase one) was set as a pre-intervention evaluation, while the music interventional measurement was set as a post-intervention evaluation (phase two). The participants conducted a non-music trial (T1), pre-exercise music trial (T2), and simultaneous music trial (T3) in a randomized order with 48–72 h apart.

### 2.2. Experimental Procedure

**Figure 1:**
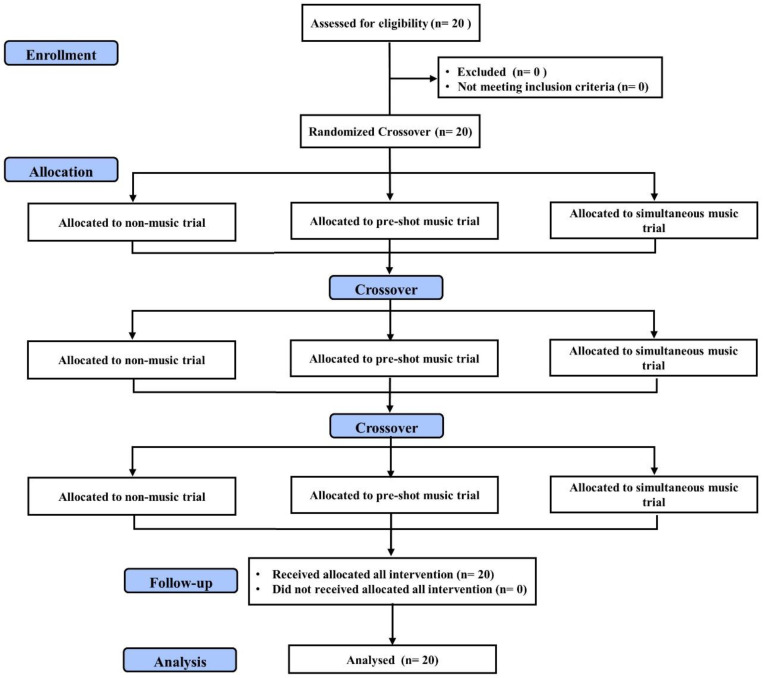
Flow diagram of the study.

The participants first visited our laboratory for determining self-selected music and familiarization of experimental procedures. Then, their height and weight were determined via a stadiometer (Seca 213, SECA, Hamburg, Germany) and an electrical weight scale (Xyfwt382, TECO, Taiwan). The participants were required to perform non-music trial, pre-exercise music trial, and simultaneous music trial (during interventional assessment) in three different occasions, 48–72 h apart. A random number program (https://www.randomizer.org/) was used to assign the order of trials for each participant (Figure 1).

**Figure 2:**
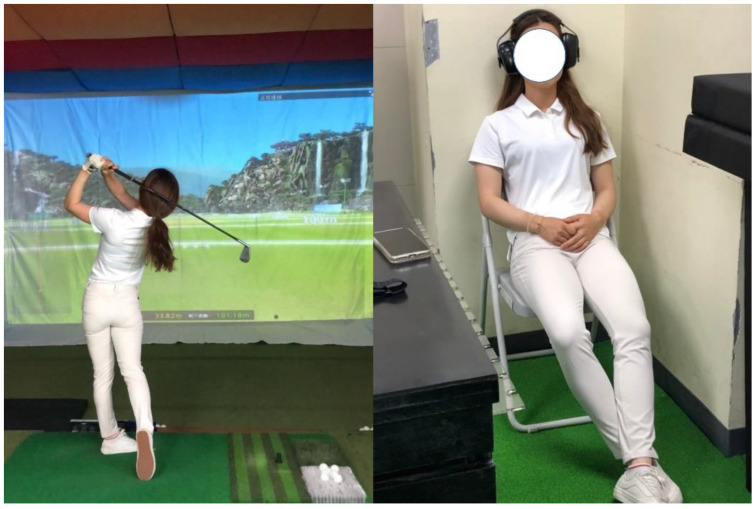
Illustration of experimental setting of golf performance and music interventions.

At the beginning of the experiment, a portable HR monitor (Polar RS800CX, Polar Electro, Kemple, Finland) was mounted at the participants’ chest level to assess the HR and HRV. For the baseline measurement, the experiment started with resting HR and HRV assessment for 10 min and reporting for the state-trait anxiety inventory-state questionnaire (STAI-S). The first 5 min of HR and HRV records were discarded due to orthostatic effect. Afterwards, the participants performed 5 shots of swing and 20 shots of putting in the Golfzon simulation training room (Vision compact golf simulator coaching system, Golfzon, Seoul, Korea) at the University of Taipei (Figure 2). During phase two assessment, the participants sat for 10 min resting or listening to pre-exercise music intervention (the participants were required to listen to self-selected music), followed by the HR and HRV measurements in a sitting position for 5 min (listening to self-selected music for T3). Afterwards, 5 shots of golf swing, 20 shots of golf putting, and the STAI-S questionnaire were measured again. In the simultaneous music trial, the participants were instructed to listen to self-selected music while completing their golf performance. The participants used their individual pre-exercise routine to complete the golf tasks. The participants used their individual 7 iron club and putter during swing and putting assessments and they were familiarized with the Golfzon simulation device through their regular training sessions. The ambient temperature and relative humidity were controlled around 20–25 °C and 50–60%, respectively. The experiment was conducted during mid-term of the university semester and all trials were conducted at the same time each day (resting day of training sessions).

### 2.3. Participants

Twenty collegiate golfers voluntarily participated in this study (age = 20.2 ± 1.4 years, height = 171.7 ± 8.0 cm, body weight = 69.5 ± 14.6 kg, golf experience = 7.5 ± 2.1 years). The inclusion criteria were: (1) collegiate golfers and (2) weekly training frequency 3–5 times a week (weekly training time of 8–10 h). The exclusion criteria were (1) report of any history of neuromuscular injury; (2) lower extremity or low-back injuries within 6 months; (3) current cardiovascular or metabolic diseases; and (4) mental health disorders such as anxiety, depression, or schizophrenia. All participants matched the inclusion criteria and finalized the assessments. The human ethical information was approved by the Institute Ethics Committee of University of Taipei (UT-IRB-2019-061). This study was performed in accordance with the ethical standards of the Institutional Human Ethical Committee and with the Declaration of Helsinki and its later amendments.

The sample size estimation was determined by using G*Power 3.1.9.4 software (G*Power, Düsseldorf, Germany). There was no relevant study design available to calculate the sample size of our study, thus a post hoc type of power analysis was used to estimate the power. A statistical test with analysis of variance (ANOVA) was completed—repeated measures within factors with an α level of 0.05 and total sample size of 20 were set to estimate the actual power (1-β error probability), which indicated that the effect size (ES) of 0.3 was required to approach the actual power of 0.81.

### 2.4. Music Intervention

The participants brought their favorite pop music to last for the 15 min duration. Individual self-selected music intervention was used during the pre-shot music trial and the simultaneous music trial. The participants sat on a comfortable chair and maintained their posture during the music intervention in a dark and quiet environment. A three position protective earmuff with a noise-reduction rating of 27 dB was used to avoid environmental noise while resting or during the music interventions (1427, 3M, China). Music was delivered via a self-prepared earlobe connected to an individual smartphone. The tempo of self-selected music was defined as slow tempo music (<120 bpm) or fast tempo music (>120 bpm) [33]. Music tempo was analyzed via BPM analyzer software (Mixmeister, Cumberland, RI, USA).

### 2.5. Golf Performance

The golf full swing and putting performance were assessed via a virtual golf simulation device (Vision Compact, Golfzon, Seoul, Korea). The Golfzon golf simulation system consists of an acquired image, T3 sensor, T3 kiosk with touch monitor, a camera, a 3000 ansi lumens projector, and a computer to evaluate the flight direction and trajectory of ball movement. The drive center mode of the golf simulator system was used to measure swinging performance. The swing performance included flight distance with carry and roll distance (flight), flight distance, speed of the ball (speed), launch angle (angle), flight direction of the ball deviation (direction), and sidespin rates of the ball (curve). In direction and curve variables, positive and negative values indicate toward right and left, respectively. In addition, constant errors (CE) and absolute errors (AE) were used to evaluate the tendency of ball direction and curve deviations during swing performance. The constant errors were calculated as average of positive and negative values. Whereas absolute values were calculated as average value of difference between direction/curve deviation and neutral value.

The putting practice mode of the golf simulator system was used to measure putting performance. The default setting of hole size was 108 mm. Putting distance was at 2.5 yards. The assessment of putting performance included the number of successful trials (putting goal) and error distance between hole and ball in unsuccessful trials (putting mean). The successful trial was defined as the golf ball reaching the golf hole.

### 2.6. Heart Rate and Heart Rate Variability

The HR and HRV were recorded via a portable HR monitor (Polar RS800CX, Polar Electro, Kemple, Finland). The raw data were analyzed via a commercial HRV analysis software (Premium version 3.2.0, Kubios, Kuopio, Finland). Moderate artefact correction and smoothing priors set at 500 lambda were used for HRV analysis [34]. The mean HR, standard deviation of normal R-R interval (SDNN) and mean sum of the squared differences between RR (RMSSD) were calculated by using the standard formulae for time domain analysis. Furthermore, the power spectra of R-R intervals were calculated by means of fast Fourier transformation for frequency domain analysis. The power of low-frequency power spectrum (LF) and high-frequency power spectrum (HF) were used to calculate the powers of frequency bands. The LF and HF spectrum ranges were set as 0.04–0.15 Hz and 0.15–0.4 Hz, respectively [35]. The LF and HF ratio was calculated to estimate sympatho-vagal balance. In addition, standard deviation of the points perpendicular to the line-of-identity (SD1) and standard deviation of along the line-of-identity (SD2) were determined to understand the nonlinear characteristics of HRV. Lastly, parasympathetic nervous system index was calculated based on meanRR, RMSSD, and SD1. Sympathetic nervous system index was calculated based on mean HR, Baevsky’s stress index, and SD2. Stress index is the result of square root of Baevsky’s stress index [36].

### 2.7. State-Trait Anxiety Inventory-State

The Chinese version of STAI-S questionnaire was used to evaluate anxiety level [37]. This questionnaire consisted of twenty statements and the final scores can be used to indicate the current anxiety level during the assessment. Each statement is scored on a four-point Likert scale (in which 1 point means not at all, 4 points mean very much so). The minimum score of STAI-S is twenty points, whereas the maximum score of STAI-S is eighty points.

### 2.8. Statistical Analyses

Descriptive data of the measured variables were presented as mean and standard deviations (mean ± SD). Percentage change in phase one and phase two was calculated as % parameter = (parameter_phase two_ − parameter_phase one_)/parameter_phase one_ × 100% [38].

The normal distribution of study variables was examined with the Kolmogorov–Smirnov test and the homogeneity was examined by using the Levene’s test (*p* > 0.05). A two-way repeated measurement ANOVA (phase (2) × trials (3)) was used to test all dependent variables, except a three-way repeated measurement of ANOVA (phase (2) × trials (3) × duration (5)) for the HR variable. When a significant interaction or main effect was identified, a post-hoc analysis with the Bonferroni contrast was used to identify the difference between the mean values. Since normal distribution of golf swing and putting performance did not pass the normality of examination, the nonparametric Wilcoxon rank test was subsequently used to identify the differences of pairwise comparisons. In addition, the ES in pairwise comparisons was tested by using the Cohen’s d standardized differences. The standardized differences of the ES were interpreted as trivial (0.0–0.2), small (0.2–0.6), moderate (0.6–1.2), large (1.2–2.0) and very large (>2.0) [39]. An alpha value of 0.05 was set for significant differences between the means (*p* < 0.05). All statistical analyses were performed by SPSS version 25.0 software for Windows (IBM, Armonk, NY, USA).

## 3. Results

### 3.1. Physical Characterstics and Individual Self-Selected Music

The results show physical profiles and tempo of individual’s self-selected music (Table 1). Thirteen participants selected slow tempo pop music [113.8 ± 8.6 bpm (95.7–119.8 bpm)] while the other seven participants selected fast tempo pop music [130.7 ± 5.2 bpm (120.2–137.4 bpm)].

Data are presented as mean and standard deviation. Slow tempo music frequency < 120 bpm; fast tempo music frequency > 120 bpm.

### 3.2. Golf Performance

There were no significant differences in all pairwise comparisons of golf performance, except in pairwise comparison in percentage change between T1 and T2 (*p* = 0.029, ES = −0.64) in angle variable (Table 2).

### 3.3. State-Trait Anxiety Inventory-State

**Figure 3:**
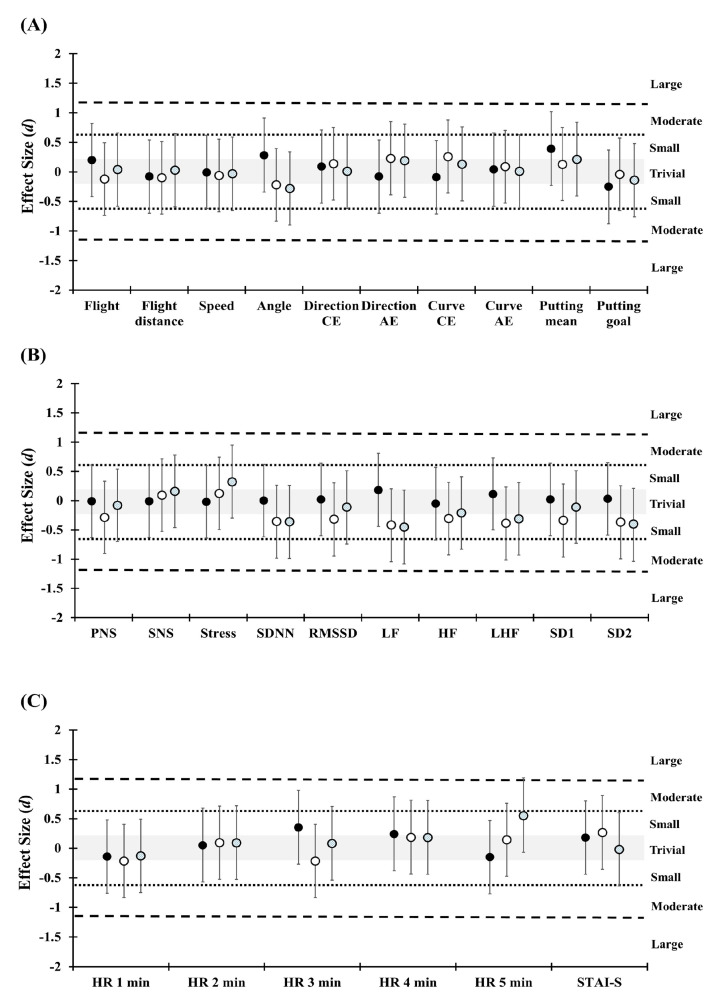
Standardized differences (effect size) of the golf swing, golf putting, heart rate variability, heart rate, and state-trait anxiety inventory-state questionnaire between phase one and phase two measurements. (**A**) notes the effect size in golf swing and putting performance; (**B**) notes the effect size in heart rate variability variables; (**C**) notes the effect size in heart rate responses and anxiety level. The positive values of effect size indicate A value greater than B value and vice versa. Grey area represents trivial effect size. The black full circle indicates phase one and phase two comparison in non-music trial. The open circle indicates phase one and phase two comparison in pre-exercise music trial. The grey circle indicates phase one and phase two comparison in simultaneous music trial. CE = constant errors; AE = absolute errors; PNS = parasympathetic nervous system index; SNS = sympathetic nervous system index; SDNN = standard deviation of normal R-R interval; RMSSD = root mean square of successive RR interval differences; LF = low frequency power spectrum; HF = high frequency power spectrum; LHF = low frequency and high frequency ratio; SD1 = the standard deviation of the points perpendicular to the line of symmetry; SD2 = the standard deviation of the points along the line of symmetry; HR = heart rate; STAI-S = state-trait anxiety inventory-state questionnaire.

**Figure 4:**
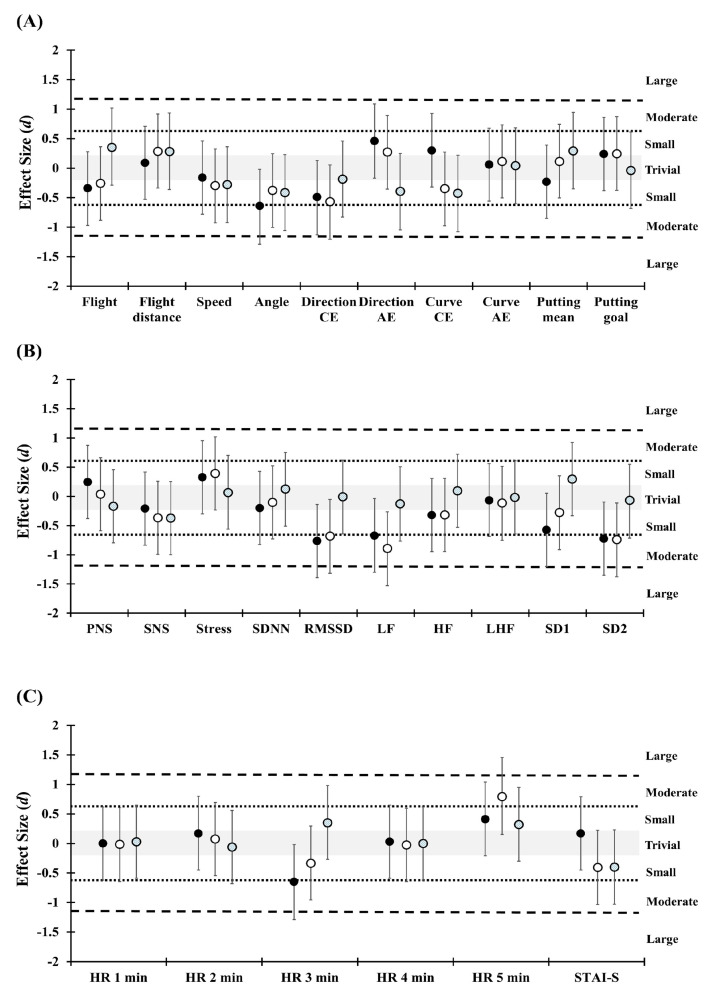
Standardized differences (effect size) of phase one and phase two percentage changes in the golf swing, golf putting, heart rate variability, heart rate, and state-trait anxiety inventory-state questionnaire between trials. (**A**) notes the effect size in golf swing and putting performance; (**B**) notes the effect size in heart rate variability variables; (**C**) notes the effect size in heart rate responses and anxiety level. The positive values of effect size indicate A value greater than B value and vice versa. Grey area represents trivial effect size. The black full circle indicates comparison between non-music trial and pre-exercise music trial. The open circle indicates comparison between non-music trial and simultaneous music trial. The grey circle indicates comparison between non-music trial and simultaneous music trial. CE = constant errors; AE = absolute errors; PNS = parasympathetic nervous system index; SNS = sympathetic nervous system index; SDNN = standard deviation of normal R-R interval; RMSSD = root mean square of successive RR interval differences; LF = low frequency power spectrum; HF = high frequency power spectrum; LHF = low frequency and high frequency ratio; SD1 = the standard deviation of the points perpendicular to the line of symmetry; SD2 = the standard deviation of the points along the line of symmetry; HR = heart rate; STAI-S = state-trait anxiety inventory-state questionnaire.

Table 2 shows the significant difference of STAI-S that was found in phase one and phase two, in comparison in the pre-exercise trial (*p* = 0.047, ES = 0.32). Significant difference of STAI-S was also found in percentage change between T2 and T3 (*p* = 0.013, ES = −0.40) (see Figure 3 and Figure 4 for ES).

### 3.4. Heart Rate and Heart Rate Variability

The results of HR responses demonstrate duration, time and trials interaction (*F* (8, 152) = 2.18, *p* = 0.032, η2 = 0.103) and duration and time interaction (*F* (4, 76) = 3.48, *p* = 0.012, η2 = 0.155). In addition, percentage change in HR responses show duration and trials interaction (*F* (8, 152) = 2.19, *p* = 0.031, η2 = 0.104) and main effect of duration (*F* (4, 76) = 3.51, *p* = 0.011, η2 = 0.156). The post hoc analysis revealed significant differences in phase comparison of HR_3min_ in T1 (*p* = 0.045, ES = 0.38) and HR_5min_ in T3 (*p* = 0.002, ES = 0.54) and trial comparison of percentage change of HR_5min_ between T1 and T3 (*p* = 0.022, ES = 0.87) (Table 3) (Figure 3 and Figure 4).

The results of HRV indices show time and trials interaction (*F* (2, 38) = 3.29, *p* = 0.048, η2 = 0.148) and main effect (*F* (1, 19) = 8.64, *p* = 0.008, η2 = 0.313) of time on SDNN. Time and trials interaction (*F* (2, 38) = 4.33, *p* = 0.020, η2 = 0.186) and main effect (*F* (1, 19) = 4.65, *p* = 0.044, η2 = 0.197) of time was found in LF. Time and trials interaction (*F* (2, 38) = 3.46, *p* = 0.042, η2 = 0.154) and main effect (*F* (1, 19) = 9.6, *p* = 0.007, η2 = 0.325) of time was also found in SD2. The post hoc analysis revealed significant differences in phase comparison of SDNN in T2 (*p* = 0.002, ES = −0.30) and T3 (*p* = 0.017, ES = −0.36). Furthermore, significant differences in phase comparison were found in RMSSD in T2 (*p* = 0.005, ES = −0.26), LF in T2 (*p* = 0.037, ES = −0.36) and T3 (*p* = 0.004, ES = −0.45), SD1 in T2 (*p* = 0.005, ES = −0.28), SD2 in T2 (*p* = 0.005, ES = −0.31) and T3 (*p* = 0.007, ES = −0.40), and stress index in T3 (*p* = 0.048, ES = 0.32). The percentage changes in HRV indices found significant difference in SDNN (*p* = 0.029, ES = −0.73) and LF (*p* = 0.034, ES = −0.64) between T1 and T2 comparison. In addition, significant differences in percentage change were found in LF (*p* = 0.018, ES = −0.80) and SD2 (*p* = 0.048, ES = −0.65) between T1 and T3 (Table 3) (Figure 3 and Figure 4).

## 4. Discussion

This was the first study to examine the acute effects of self-selected music intervention on golf swing, golf putting, HR, HRV, and anxiety. The main purpose of this study was to examine the acute effects of pre-exercise and simultaneous self-selected music interventions on golf swing and putting performance in collegiate golfers. The secondary purpose was to determine anxiety, HR, and HRV during pre-task and simultaneous music tasks. The outcomes of measurement rejected our first hypothesis. The findings of the study show no difference of golf swing and putting performance when pre-exercise and simultaneous self-selected music interventions were implemented to collegiate golfers. The results in our study only reveal that the ball launch angle was different between non-music and pre-exercise music trials. However, the secondary hypothesis was not rejected. The anxiety level was significantly reduced while parasympathetic HRV indices were augmented when a pre-task self-selected music was implemented.

Initially, the present study demonstrated no significant effects of pre-exercise and simultaneous self-selected music interventions on golf performance in a simulating environment. Specifically, only ball launch angle revealed significant differences between non-music trial and pre-exercise music trial. These findings indicate that music intervention has a trivial effect on task engagement in closed-chain motor skills, as demonstrated by golf performance. Interestingly, music stimuli can increase motor neural activities [6,23,24], cardiovascular functions [32], and perceptual and cognitive responses [19,40] during exercise. Pre-task music intervention has shown beneficial effects on anaerobic-based short-term bouts of exercise but not on aerobic-based prolonged exercise [27]. These benefits were observed while engaging gross motor skills. In contrast, Arbinaga et al., [41] demonstrated that there were no benefits of slow-tempo (60 bpm) and fast-tempo (105 bpm) music interventions on dart-throwing performance (closed chain motor skills) in female university students. Arbinaga’s study might be limited by using non-preferred music intervention, which might not cause significant impact to exercise motivation. Our findings further support the notion that music intervention contributes no or minor benefits to the execution of the fine motor tasks in experienced amateur collegiate golfers.

Simultaneous music has a unique feature that transforms music tempo as concurrent feedback for the coordination of movement. Tapps et al., [29] reported that the synchronized music intervention improved putting performance in American collegiate golfers, regardless of the type of music. However, this observation was not supported in our findings. The controversial finding was potentially related to the discrepancy of putting the task between studies. For example, Tapps et al. conducted the experiment in a putting green and listened to multiple types of music whereas the participants in our study performed golf putting in a simulate environment and listened to self-selected music. A semi-structured interview depicted the popularity of using music intervention in amateur and semi-professional golfers, given the profound use of self-selected music in golfers [30]. The advantage of using self-selected music is related to individual characteristics of cognition in association with motor behaviors [31]. However, lack of performance enhancement found in the current study could challenge integration with preferable music during regular golf practice.

In term of psychological performance, the result demonstrates a positive effect of the pre-exercise music intervention on anxiety level. In the phase one and phase two comparison, the decrease in STAI-S scores (35.2 points to 32.8 points) found in the pre-exercise music trial indicates the advantage of using music as a pre-event routine. The underlying mechanisms to alleviate anxiety level is related to change in cerebral function via the limbic system and consequently results in psychological relaxation and the feeling of pleasure [42]. Thus, music-induced cerebral activities is suggested as a major mechanism to alleviate anxiety and neurophysiological responses [23]. Our findings in the pre-exercise music trial was in agreement with a qualitative study that showed improvement in self-confidence and relief of psychological strains after a music intervention in amateur golfers [30]. However, the simultaneous music trial did not predominate anxiety levels in the current study. This finding was associated with a recent study that showed no influence of simultaneous music on anxiety status while measuring dart-throwing performance in female university students [41]. The discrepancy of psychological benefits between pre-exercise music and simultaneous music depicted the specific nature of mental readiness in relation to golf tasks. Nevertheless, our findings support the aspects of music intervention in exercise health, and sports sciences may consider adopting psychological skills to control anxiety levels for pre-event preparation.

In view of autonomic function, pre-exercise and simultaneous music interventions showed minimal impact on the HR regulation. In contrast, we observed significant changes in HRV metrics such as SDNN, LF, and SD2 when music intervention was applied. Increase in SDNN and SD2 variables indicated an increase in parasympathetic outflow elicited by music interventions. Furthermore, augment of LF indices in music trials indicated concurrent change of sympatho-vagal activation. The mechanisms that cause similarity of HR responses and variety of HRV modulation among the music trials is unclear in the current study. It might be assumed that a music-induced emotional process plays a role to cause the difference in the HR and HRV activation in our study [32]. The underlying mechanisms to regulate the cardiac-autonomic activities during golf performance need to be examined by future studies.

The first limitation of this study is that the experimental condition was unable to be blinded to the participants. Thus, psychological factors might limit our findings. The second limitation is that the participants completed the experimental conditions without vital stressors, compared to a game situation. Similarity of research outcomes in golf performance among the trials could potentially relate to non-competent factors in the experiment. Future studies are suggested to investigate effects of different types of music intervention on golf performance under versatile mental stresses. The third limitation is that the outcomes of the present study could not be used to integrate chronic effects of pre-exercise or simultaneous music intervention. Music-based intervention is considered as an alternative tool for rhythmic training to improve time-control of human movement [43]. Investigation on chronological effects of music intervention on dynamic features of real-time golf performance in future studies is recommended.

Despite the advantages of concurrent music intervention on psychophysiological modulation, the forbidden nature of using electronic devices during sports competition may not permit the implementation of simultaneous music intervention for athletes. This restriction particularly restricts golfers from using music devices while playing golf. However, a rhythmic technique with metronome devices could be critical to overcoming psychological strains during golf competitions. Previous studies have shown that simultaneous metronome training can improve golf shot accuracy and decrease the variability of swing performance [44,45]. Another example is that hand and feet tapping training improves accuracy of swing performance [46]. Nevertheless, utilization of simultaneous music intervention may be an optimal alternative to improve rhythmic technique for the timing control of golf shots during training contexts.

## 5. Conclusions

In conclusion, there is no prominence of the benefits of music on golf swing and putting performance during pre-exercise and simultaneous self-selected music tasks in collegiate golfers. The pre-exercise self-selected music intervention has a positive effect on the reduction in anxiety level. The positive benefits of pre-exercise self-selected music intervention on cardia-related modulation revealed in the current study predominate psychophysiological contributions during golf performance. Our study further explored empirical evidence on psychophysiological benefits for music use during regular training sessions in collegiate golfers.

## Figures and Tables

**Table 1:** Physical characteristics of participants.

Variables	Mean ± SD
Gender (male/female, n)	15/5
Age (years)	20.2 ± 1.4
Height (cm)	171.7 ± 8.0
Weight (kg)	69.5 ± 14.6
Golf experience (years)	7.5 ± 2.1
Music tempo (bpm, fast/slow)	130.7 ± 5.2/113.8 ± 8.6

**Table 2:** Descriptions of golf swing, golf putting, and anxiety level in non-music, pre-exercise music, and simultaneous music trials.

Variables	Non-Music Trial (T1)		Pre-Excise Music Trial (T2)		Simultaneous Music Trial (T3)		T1 Phase One/Two % Difference	T2 Phase One/Two % Difference	T3 Phase One/Two % Difference
	Phase One	Phase Two	Phase One	Phase Two	Phase One	Phase Two			
Flight (m)	162.1 ± 23.7	155.2 ± 41.9	164.4 ± 23.3	166.5 ± 22.6	162.8 ± 19.3	161.9 ± 23.1	−4.0 ± 21.9	1.4 ± 4.3	−0.8 ± 5.6
Flight distance (m)	147.5 ± 25.8	149.5 ± 24.9	151.6 ± 22.7	153.2 ± 22.5	149.4 ± 20.2	148.8 ± 24.3	1.8 ± 8.2	1.2 ± 4.1	−0.8 ± 6.5
Speed (m·s^−1^)	76.7 ± 34.9	76.8 ± 34.3	87.7 ± 31.8	88.5 ± 31.9	81.4 ± 35.5	82.4 ± 34.7	0.6 ± 4.7	1.3 ± 4.5	3.5 ± 16.0
Angle (degree)	19.3 ± 5.1	18.1 ± 3.1	18.2 ± 3.3	18.9 ± 3.9	17.1 ± 4.9	18.2 ± 2.3	−3.6 ± 14.2	3.6 ± 6.0 #	152.7 ± 675.5
Direction CE (degree)	0.3 ± 2.1	0.1 ± 2.1	0.7 ± 2.7	0.2 ± 2.2	0.3 ± 2.1	0.3 ± 1.9	−207.0 ± 499.6	−21.0 ± 156.0	−2.3 ± 236.1
Direction AE (degree)	2.0 ± 1.4	2.1 ± 1.2	2.3 ± 1.7	2.0 ± 1.1	2.1 ± 1.4	1.9 ± 1.0	68.3 ± 200.7	0.3 ± 49.0	18.4 ± 71.6
Curve CE (bpm)	75.4 ± 395.7	111.0 ± 358.8	−144.3 ± 366.1	−246.2 ± 313.3	−62.3 ± 421.9	−120.0 ± 424.2	−95.2 ± 324.8	−1374.2 ± 5830.9	−10.1 ± 235.0
Curve AE (bpm)	428.0 ± 265.3	417.8 ± 210.8	448.2 ± 273.0	419.6 ± 191.1	425.8 ± 224.6	423.8 ± 236.7	29.1 ± 110.4	23.4 ± 88.4	13.9 ± 56.8
Putting mean (yards)	0.4 ± 0.2	0.3 ± 0.2	0.3 ± 0.2	0.3 ± 0.2	0.3 ± 0.2	0.3 ± 0.2	−1.7 ± 84.7	23.3 ± 126.1	−15.5 ± 71.4
Putting goal (n)	12.1 ± 3.0	13 ± 3.7	13.3 ± 3.7	13.4 ± 3.7	13.1 ± 2.8	13.6 ± 4.2	15.0 ± 42.8	5.7 ± 34.0	4.0 ± 27.0
STAI-S	34.4 ± 8.8	33 ± 6.8	35.2 ± 7.6	32.8 ± 7.6 *	34.7 ± 9.6	34.9 ± 9.3	−2.9 ± 8.7	−5.5 ± 19.0	1.6 ± 16.0 †

T = trial; CI = confident interval; CE = constant errors; AE = absolute error; STAI-S = state-trait anxiety inventory-state questionnaire; m = meter; n = number; ms = microsecond; n = numbers. * indicates significant difference between phases. # indicates significant difference in comparison with non-music trial. † indicates significant difference in comparison with pre-exercise music trial. In direction and curve variables, positive and negative values indicate toward right and left, respectively.

**Table 3:** Descriptions of heart rate and heart rate variability in non-music, pre-exercise music, and simultaneous music trials.

Variables	Non-Music Trial (T1)		Pre-Excise Music Trial (T2)		Simultaneous Music Trial (T3)		T1 Phase One /Two % Difference	T2 Phase One /Two % Difference	T3 Phase One /Two % Difference
	Phase One	Phase Two	Phase One	Phase Two	Phase One	Phase Two			
SDNN (log)	3.6 ± 0.3	3.6 ± 0.4	3.5 ± 0.5	3.6 ± 0.5 *	3.5 ± 0.4	3.7 ± 0.4 *	−0.1 ± 7.7	4.7 ± 5.2 #	4.6 ± 7.9
RMSSD (log)	3.3 ± 0.5	3.3 ± 0.5	3.3 ± 0.6	3.4 ± 0.6 *	3.3 ± 0.5	3.4 ± 0.6	0 ± 10.4	5.6 ± 9.7	2.1 ± 11.4
LF (log)	6.7 ± 0.7	6.6 ± 0.8	6.2 ± 0.9 #	6.6 ± 1.1 *	6.3 ± 0.9	6.7 ± 0.8 *	−1.7 ± 11.8	5.9 ± 11.6 #	7.0 ± 9.2 #
HF (log)	5.6 ± 0.9	5.7 ± 1.2	5.5 ± 1.2	5.8 ± 1.3	5.5 ± 1.0	5.7 ± 1.1	1.3 ± 16.6	7.1 ± 21.8	4.9 ± 14.2
LHF (log)	4.0 ± 3.6	3.6 ± 3.8	2.4 ± 1.4	3.2 ± 3.0	3.0 ± 2.1	4.1 ± 4.5	34 ± 136.8	38.7 ± 123.1	37.8 ± 84.3
SD1 (log)	3.0 ± 0.5	3.0 ± 0.5	2.9 ± 0.6	3.1 ± 0.6 *	3.0 ± 0.5	3.0 ± 0.6	0 ± 11.6	6.3 ± 11.3	2.4 ± 12.9
SD2 (log)	3.9 ± 0.3	3.9 ± 0.4	3.7 ± 0.5	3.8 ± 0.5 *	3.7 ± 0.4	3.9 ± 0.4 *	−0.1 ± 7.1	4.3 ± 5.4	4.6 ± 7.0 #
PNS index	−1.2 ± 0.8	−1.2 ± 0.8	−1.0 ± 1.0	−0.8 ± 1.0	−1.1 ± 0.9	−1.0 ± 1.1	−4.5 ± 95.1	−27.6 ± 70.9	−15.6 ± 92.9
SNS index	1.6 ± 1.2	1.6 ± 1.4	1.6 ± 1.5	1.3 ± 1.8	1.6 ± 1.3	1.4 ± 1.4	−111.5 ± 447.3	−52.5 ± 97.3	−17.7 ± 104.6
Stress index	12.6 ± 3.7	12.6 ± 5	13.6 ± 5	12.6 ± 6.5	13.8 ± 5.1	12.2 ± 4.5 *	1.7 ± 27.7	−7.8 ± 25.4	−10.0 ± 20.3
HR_1mn_ (log)	4.4 ± 0.1	4.4 ± 0.1	4.4 ± 0.1	4.4 ± 0.2	4.4 ± 0.1	4.4 ± 0.1	0.5 ± 2.5	0.5 ± 2.3	0.5 ± 1.7
HR_2mn_ (log)	4.4 ± 0.1	4.4 ± 0.1	4.4 ± 0.1	4.3 ± 0.2	4.4 ± 0.1	4.4 ± 0.2	−0.1 ± 2.3	−0.7 ± 3.0	−0.5 ± 2.4
HR_3mn_ (log)	4.4 ± 0.1	4.4 ± 0.1 *	4.4 ± 0.1	4.4 ± 0.2	4.4 ± 0.1	4.4 ± 0.2	−1.1 ± 2.2	0.5 ± 2.4	−0.4 ± 2.5
HR_4mn_ (log)	4.4 ± 0.1	4.4 ± 0.1	4.4 ± 0.1	4.4 ± 0.2	4.4 ± 0.1	4.3 ± 0.2	−0.8 ± 2.8	−0.8 ± 2.4	−0.9 ± 2.5
HR_5mn_ (log)	4.4 ± 0.1	4.4 ± 0.1	4.4 ± 0.1	4.3 ± 0.2	4.4 ± 0.1	4.3 ± 0.2 *	0.5 ± 3.1	−1.1 ± 4.1	−1.9 ± 2.3 #

T = trial; PNS = parasympathetic nervous system index; SNS = sympathetic nervous system index; SDNN = standard deviation of normal R-R interval; RMSSD = root mean square of successive RR interval differences; LF = low frequency power spectrum; HF = high frequency power spectrum; LHF = low frequency and high frequency power spectrum ratio; SD1 = the standard deviation of the points perpendicular to the line of symmetry; SD2 = the standard deviation of the points along the line of symmetry; ms = microsecond; log = natural logarithm; bpm = beats per minute. * indicates significant difference between phases. # indicates significant difference in comparison with non-music trial.
